# Management of primary chronic headache in the general population: the Akershus study of chronic headache

**DOI:** 10.1007/s10194-011-0391-8

**Published:** 2011-10-13

**Authors:** Espen Saxhaug Kristoffersen, Ragnhild Berling Grande, Kjersti Aaseth, Christofer Lundqvist, Michael Bjørn Russell

**Affiliations:** 1 Head and Neck Research Group, Research Centre, Akershus University Hospital, 1478 Lørenskog, Oslo, Norway; 2 General Practice Research Unit, Department of General Practice, Institute of Health and Society, University of Oslo, Oslo, Norway; 3 Institute of Clinical Medicine, Akershus University Hospital, University of Oslo, Nordbyhagen, Norway; 4 HØKH, Research Centre, Akershus University Hospital, Lørenskog, Oslo, Norway

**Keywords:** Primary chronic headache, Chronic migraine, Medication-overuse headache, Health care utilisation, General population

## Abstract

Primary chronic headaches cause more disability and necessitate high utilisation of health care. Our knowledge is based on selected populations, while information from the general population is largely lacking. An age and gender-stratified cross-sectional epidemiological survey included 30,000 persons aged 30–44 years. Respondents with self-reported chronic headache were interviewed by physicians. The International Classification of Headache Disorders was used. Of all primary chronic headache sufferers, 80% had consulted their general practitioner (GP), of these 19% had also consulted a neurologist and 4% had been hospitalised. Co-occurrence of migraine increased the probability of contact with a physician. A high Severity of Dependence Scale score increased the probability for contact with a physician. Complementary and alternative medicine (CAM) was used by 62%, most often physiotherapy, acupuncture and chiropractic. Contact with a physician increased the probability of use of CAM. Acute headache medications were taken by 87%, while only 3% used prophylactic medication. GPs manage the majority of those with primary chronic headache, 1/5 never consults a physician for their headache, while approximately 1/5 is referred to a neurologist or hospitalised. Acute headache medication was frequently overused, while prophylactic medication was rarely used. Thus, avoidance of acute headache medication overuse and increased use of prophylactic medication may improve the management of primary chronic headaches in the future.

## Introduction

Headache is a common complaint in the general population. The personal burden, social impact and economic cost for both the sufferer and society are significant [1]. The International Classification of Headache Disorders (ICHD-II) divides headaches into primary and secondary forms [2]. The most common primary headaches are migraine and tension-type headaches, while other primary headaches are rare [3]. The primary headaches are usually paroxysmal, but 3% of the general population has primary chronic headache, i.e. more than 15 headache days per months [4].

Headache accounts for 4% of the general practitioners (GPs) consultations, and 2–4% of these are referred to specialists or hospitals [5, 6]. Headache is probably the most common reason for referral to neurologists [5–7]. Approximately 20–30% of all new referrals to out-patients neurological departments are due to headache [5, 6, 8, 9].

Our aim was to investigate primary chronic headache in the general population in order to evaluate utility of health services and medication use, since this knowledge is generally lacking.

## Methods

### General design

**Fig. 1:**
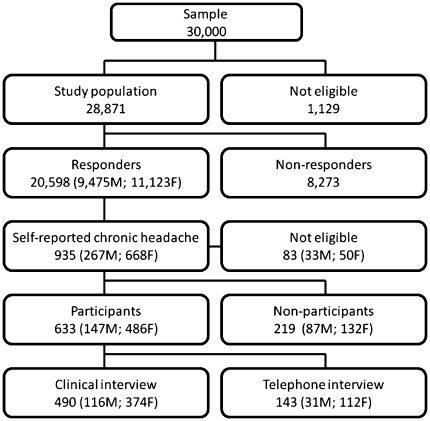
Flow chart of the participation

This is a cross-sectional epidemiological survey. A short postal questionnaire screened for chronic headache (≥15 days/last month and/or ≥180 days/last year). Participants with self-reported chronic headache were invited to a clinical interview, a physical and neurological examination conducted by neurological residents. The response rate of the screening questionnaire was 71% and the participation rate of the interview was 74%. Figure 1 shows a flow chart of the study. The method has been described in more detail elsewhere [4].

### Setting

The study was conducted in 2005 at Akershus University Hospital, Oslo, Norway. Provision of health care in Norway is based on a decentralized model. The state is responsible for policy design, overall capacity and quality of health care and hospital services. Almost all general practices are private enterprises and their services are contract based. The GPs are reimbursed through a fixed annual fee, and fees for the specific services from the National Health Insurance and the patients. Each Norwegian citizen has through legislation the right to be on a GPs list, and it is possible to change GP twice a year. The GP is the gate keeper for referral to specialists and hospitals except in emergencies.

### Participants

A random sample of 30,000 persons aged 30–44 years and stratified for age and gender was drawn from the general population of eastern Akershus County, Norway. The area includes rural and urban municipalities in close proximity to Oslo.

### Headache classification

The headaches were classified according to explicit diagnostic criteria of the ICHD-II and its relevant revisions, i.e. the latest update was used in relation to chronic migraine and medication overuse headache [2, 10–12]. Primary chronic headache was defined as headache at least 15 days per months for at least 3 months, not secondary to a head trauma, brain tumour, etc. Those with medication overuse as defined by ICHD-II were included as primary chronic headache [2].

### Physician consultation

We defined four levels of contact due to headache, i.e. none (no physician contact), primary (GP), secondary (neurologist) and tertiary (hospitalisation). A GP referral is a prerequisite for access to neurologists, while both GP and neurologist can refer to the hospital.

### Complementary and alternative contacts

The complementary and alternative medicine (CAM) forms queried were acupuncture, chiropractic, homeopathy, naprapath, physiotherapy, psychologist and psychomotor physiotherapy.

### Medication use and dependency

We asked about current medication use, and excluded medication used for other pain conditions. To assess dependency like behaviour, we used the The Severity of Dependence Scale (SDS), which includes five questions designed to measure psychological dependence (Table 1) [13]. The questions apply to the headache medication taken within the last month. Each item is scored on a 4-point scale (0–3), and the total maximum score is 15. The method has been described in detail elsewhere [14].

**Table 1:** The five questions of the Severity Dependence Scale (SDS) adapted for headache medication

1	Do you think your use of headache medication was out of control? (never/almost never = 0, sometimes = 1, often = 2, always/nearly always = 3)
2	Did the prospect of missing a dose make you anxious or worried? (scoring as for question 1)
3	Did you worry about your use of your headache medication? (scoring as for question 1)
4	Did you wish you could stop? (scoring as for question 1)
5	How difficult would you find it to stop or go without your headache medication? (not difficult = 0, quite difficult = 1, very difficult = 2, impossible = 3)

“Your headache medication” was in the interview replaced with the name of the individually relevant headache medication. Each item is scored on a 4-point scale (0–3), and the total maximum score is 15

### Statistics

Data from the interviews were directly entered using SPSS Data Entry 4.0 (SPSS Inc., Chicago, IL, USA) and statistical analyses were performed using SPSS 15.00 for Windows. For descriptive data, proportions, means and confidence intervals (CI) are given. Pearson *χ* ^2^ test was used for testing significance of group differences for categorical data, Fisher’s exact test was used when appropriate. Student’s *t* test was used for numerical data. Significance levels were set at *p* < 0.05 and 95% CI were calculated. CI and probabilities are not given when *n* < 5.

### Ethical issues

The Regional Committee for Medical Research Ethics and the Norwegian Social Science Data Services approved the study. All participants gave informed consent.

## Results

### Participants and headache diagnoses

A total of 405 participants (22% men and 78% women) had primary chronic headaches. Ninety-five percent (*n* = 384) had chronic tension-type headache (CTTH), 4% (*n* = 15) had chronic migraine (CM), and 2% had other primary chronic headache, i.e. new daily persistent headache (*n* = 4) and chronic cluster headache (*n* = 1). Forty-nine percent (*n* = 199) had co-occurrence of migraine and 46% (*n* = 185) had medication overuse. The diagnoses are not mutually exclusive.

### Physician consultation pattern

Table 2 shows the physician and CAM contact pattern. Twenty percent (79/405) had never consulted their GP because of headache, while 80% (326/405) had consulted their GP. The GP referred 1/4 with primary chronic headache to neurologist and 4% had been hospitalised. Significantly more women than men had had contact with their GP (83 vs. 73%, *p* = 0.044), while referral was not influenced by gender.

**Table 2:** Contact and treatment pattern in relation to different primary chronic headache diagnoses

	CTTH without medication overuse (*N* = 216) % (*n*)	CTTH with medication overuse (*N* = 169) % (*n*)	Chronic migraine without medication overuse (*N* = 3) % (*n*)	Chronic migraine with medication overuse (*N* = 14) % (*n*)	Other primary chronic headache without medication overuse (*N* = 3) % (*n*)	Other primary chronic headache with medication overuse (*N* = 7) % (*n*)	All primary headaches (*N* = 405) % (*n*)
Contact level							
None	22 (47)	17 (28)	0 (0)	14 (2)	33 (1)	14 (1)	20 (79)
Primary	78 (169)	83 (141)	100 (3)	86 (12)	67 (2)	86 (6)	80 (326)
Secondary	16 (34)	20 (33)	33 (1)	36 (5)	67 (2)	71 (5)	19 (76)
Tertiary	4 (9)	4 (7)	0 (0)	0 (0)	33 (1)	0 (0)	4 (17)
Complementary and alternative medicine							
Acupuncture	30 (65)	34 (58)	0 (0)	57 (8)^d^	67 (2)	43 (3)	33 (133)
Chiropractic	29 (62)	28 (47)	0 (0)	29 (4)	33 (1)	14 (1)	28 (113)
Homeopathy	11 (23)	10 (17)	0 (0)	14 (2)	33 (1)	29 (2)	11 (44)
Naprapath	6 (13)	4 (6)	0 (0)	0 (0)	33 (1)	0 (0)	5 (20)
Physiotherapy	50 (109)	56 (94)	0 (0)	57 (8)	33 (1)	43 (3)	52 (211)
Psychologist	2 (4)	2 (3)	0 (0)	0 (0)	33 (1)	0 (0)	2 (8)
Psychomotor physiotherapy	6 (14)	10 (17)	0 (0)	0 (0)	0 (0)	0 (0)	8 (31)
Any CAM	61 (132)	65 (110)	0 (0)	64 (9)	67 (2)	57 (4)	62 (253)
Medication use							
Acute medication	75 (163)^b^	100 (169)^a,c^	100 (3)	100 (14)	100 (3)	100 (7)	87 (352)
Prophylactic medication	3 (7)	3 (5)	0 (0)	7 (1)	0 (0)	14 (1)	3 (14)

Individual diagnoses are not mutually exclusive

^a^ *p* < 0.001 for CTTH with medication overuse versus CTTH without medication overuse

^b^ *p* < 0.001 for CTTH without medication overuse versus all other primary headaches

^c^ *p* < 0.001 for CTTH with medication overuse versus all other primary headaches

^d^ *p* < 0.05 for chronic migraine with medication overuse versus all other primary headache

### Complementary and alternative medicine

CAM was used by 62% (253/405) (Table 2). Physiotherapy, acupuncture and chiropractic were most frequently used. The use of CAM was significantly higher among those who had consulted a physician compared to those who had not such contact (*p* < 0.001). Of those who did not consult a physician 30% had used CAM to treat their headache. Significantly more women than men had used CAM (67 vs. 48%, *p* = 0.002), physiotherapy being the only subgroup with a significant difference (57% women vs. men 36%, *p* = 0.001).

### Co-occurrence of migraine

Table 3 shows that co-occurrence of migraine as compared to no co-occurrence of migraine significantly increased the physician contact (*p* < 0.001), while referral to hospital was not significantly different in the two groups.

**Table 3:** Contact and treatment pattern for participants with primary chronic headaches

	All primary headaches without co-occurrence of migraine (*N* = 206) % (*n*)	All primary headaches with co-occurrence of migraine (*N* = 199) % (*n*)	All primary headaches without medication overuse (*N* = 220) % (*n*)	All primary headaches with medication overuse (*N* = 185) % (*n*)
Contact level				
None	28 (57)	11 (22)^a^	22 (48)	17 (31)
Primary	72 (149)	89 (177)^a^	78 (172)	83 (154)
Secondary	15 (31)	23 (45)^a^	16 (36)	22 (40)
Tertiary	5 (10)	4 (7)	5 (10)	4 (7)
Complementary and alternative medicine				
Acupuncture	28 (57)	38 (76)^b^	30 (67)	36 (66)
Chiropractic	26 (54)	30 (59)	29 (63)	27 (50)
Homeopathy	10 (20)	12 (24)	11 (24)	11 (20)
Naprapath	5 (10)	5 (10)	6 (14)	3 (86)
Physiotherapy	47 (97)	57 (114)^b^	50 (110)	55 (101)
Psychologist	2 (4)	2 (4)	2 (5)	2 (3)
Psychomotor physiotherapy	7 (14)	9 (17)	6 (14)	9 (17)
Any CAM	58 (120)	67 (133)	61 (134)	64 (119)
Medication use				
Acute medication	81 (167)	93 (185)^a^	76 (167)	100 (185)^c^
Prophylactic medication	1 (3)	6 (11)^b^	3 (7)	4 (7)
Co-occurrence of migraine	0 (0)	100 (199)	42 (92)	58 (107)^c^

Individual contacts and treatments are not mutually exclusive

^a,b^ *p* < 0.001 and *p* < 0.05, respectively, for all primary chronic headaches with versus without migraine

^c^ *p* < 0.01 for all primary chronic headaches with versus without medication overuse

The overall use of CAM was not influenced by migraine, but significantly more with than without co-occurrence of migraine had acupuncture and physiotherapy (*p* < 0.05).

### Co-occurrence of medication overuse

Medication overuse neither influenced the consultation pattern nor CAM. However, significantly more with than without medication overuse had co-occurrence of migraine (*p* = 0.001).

### Use of medication

Acute medication was used by 87, and 9% used it on a daily basis. A higher proportion of participants with than without co-occurrence of migraine used acute medication (93 vs. 81%, *p* < 0.001). More women than men used acute medication (89 vs. 79%, *p* = 0.009). Participants using acute medication had significantly more physician contact than participants not using acute medication (82 vs. 18%, *p* = 0.035).

Simple analgesic such as paracetamol (acetaminophen) and ibuprofen were most frequently overused 62% (*n* = 115), followed by combination analgesics 28% (*n* = 51). Triptans, ergotamines, opioids and combination of acute medications were overused by 10% (*n* = 10). Co-occurrence of migraine did not influence which drugs that were overused except that triptans were only overused by those with co-occurrence of migraine. Only 4% used prophylactic treatment, with significantly more use in participants with than without co-occurrence of migraine (6 vs. 2%, *p* = 0.03).

**Fig. 2:**
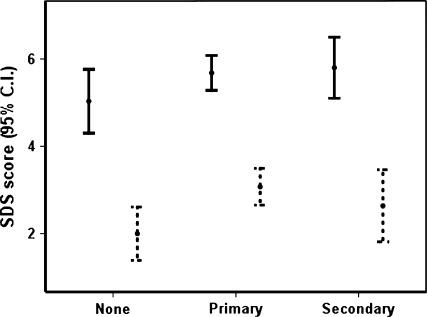
Severity Dependence Scale (SDS) scores in participants with primary chronic headache with (*black*) or without (*dashed*) medication overuse versus contact level

The SDS score was significantly higher in those with than without physician contact [4.3 (95% CI 4.0–4.6) vs. 3.2 (95% CI 2.6–3.8)], as well as in those with than without medication overuse for all levels of physician contact (Fig. 2).

## Discussion

### Presentation of main findings

Our main finding is that the majority of primary chronic headache sufferers (80%) consulted their GP due to their headache, while only 20% consulted a neurologist. Co-occurrence of migraine and a high SDS score increased the physician consulting rate. The use of CAM was high (62%) and higher if a physician had been consulted than not. Prophylactic headache medication was rarely used.

### Methodological considerations

The sample size was chosen to ensure adequate numbers of participants with chronic headache for the accurate descriptive statistics. The large sample and high response rates should ensure representative data from the general population.

The age range of patients of 30–44 years was chosen in order to target the highest number of participants with “pure” primary chronic headache, as the prevalence is lower in younger people, and older people have a higher risk of co-morbidity with other disorders. Furthermore, data from the Norwegian prescription registry indicate that the drug prescription pattern is different in people below and above 50 years of age [15].

Two neurological residents with experience in headache diagnostics conducted all interviews. The different headache diagnoses were equally frequently set by both physicians, suggesting that inter-observer variation was low. The headache diagnoses were equally frequent in participants interviewed at the clinic and in those interviewed by telephone.

Our data on medication use and health care utilisation are based on self-reports and therefore open to recall bias, although there is no reason to suspect systematic bias. Health registry data are, however, often incomplete and not necessarily more precise. In addition, registry data do not exist for a majority of treatment contacts outside traditional conventional medicine.

### Physician contact

Twenty percent of the subjects with primary chronic headache had never consulted a physician due to headache. The reason for this is unknown, but a possible reason may be due to the low status of headache. For some people, the chronic headache may represent just a minor problem in a co-morbidity of other pain condition [5, 16]. It may also be that some of those not consulting a physician prefer CAM as a treatment option or they might manage their headache adequately by themselves and do not feel a need for further help. In a study conducted in a primary care population, 28% of those with chronic headache reported that they did not need a consultation for their headache [17]. Thus, this figure corresponds well with our figure. Eighty percent consulted their GP for their primary chronic headache, and 1/4 was referred to a neurologist. The referral rate to neurologists is quite low considering that the management of chronic headaches is a real challenge that is often not successful in primary care. Other population-based studies on chronic headache found that 40–60% consulted their GP and 13–28% was referred to a neurologist during the previous 6–12 months [18–20].

In our study, 4% had been admitted to hospital in-patient treatment due to their headache. This is similar to a European general population study [18], but low compared to a US study [20]. The latter might be accounted for by medication overuse headache primarily being caused by analgesics and triptans in Europe, while barbiturates and opioids are major challenges which often require hospitalisation in medication overuse headache in USA.

Co-occurrence of migraine increased the consultation rate. Similarly, migraine caused a higher consultation rate than tension-type headache in the general Danish population [21, 22]. However, a French and an American study found the consultation rate was not influenced by co-occurrence of migraine among those who had chronic headache [18, 20].

### Use of complementary and alternative medicine

Over 60% of those with primary chronic headache had, at some point, used CAM because of their headache. Other studies have found that 40–90% of chronic headache sufferers from different clinical settings use CAM for their headache [23–25]. This indicates that chronic headache sufferers like other chronic pain sufferers are likely to use CAM as treatment [26, 27].

One reason for the high use of CAM despite the wide range of traditional medical treatments for headache, may be failure to achieve optimal control with medication.The use of CAM is high and increasing in Norway and worldwide [28, 29] and approximately 1/3 person in the Norwegian general population who use CAM use it because of headache [30]. A survey conducted in the UK among CAM providers confirmed that headache is one of the conditions believed to benefit mostly from different CAM subtypes [31].

### Use of medication

Eighty-seven percent used any acute medication for their primary chronic headache, which is similar to the 84% found in France [18]. Forty-six percent of those with primary chronic headache overused medication, which is a high proportion compared to the 25–35% found in three other population studies [19, 32, 33]. Part of this might be explained by the use of different diagnostic criteria, i.e. ICHD II versus criteria suggested by Silberstein [34, 35]. The distribution of type of overused medication is comparable to other population-based studies where simple analgesics are most frequently overused and paracetamol the main overused drug [18, 20, 33]. Paracetamol (acetaminophen) and ibuprofen are available as over-the-counter (OTC) drugs and are the most commonly used simple analgesics in Norway.

The prevalence of medication overuse may also be difficult to compare across studies using different data collection methods. The condition may, in some settings, be underreported.

The proportion of medication overuses among chronic headache sufferers in the general population are lower than that seen in headache clinics, and the spectrum of overused medication differs slightly [18, 32, 33, 36–38].

In US it has been found that 23% of chronic headache used acute medication on a daily basis [20] in contrast to only 9% in the Norwegian general population. Norwegian results comparable to those from the US are only found in neurology out-patient settings [39].

The rare use of prophylactic treatment is surprising considering the diagnosis of primary chronic headache and the frequent use of acute headache medication.

The SDS was significantly higher in those with than without medication overuse for all levels of physician contact (Fig. 2). Primary chronic headache participants in contact with physicians had significantly higher SDS than those without such contact. Thus, our results support the hypothesis that persons with physician contact differ from those without such contact, and that people with physician contact are likely to be more severe headache sufferers with more disability, dependency-like characteristics and other headache-related problems.

To alleviate the world wide problem of medication overuse headache, it is important to educate those with medication overuse headache to reduce their medication consumption. We have previously described that a short advice can reduce the medication use considerable and this also leads to the reduction of the headache frequency [40].

## Conclusion

Primary chronic headache is most often treated by health professionals, though 1/5 never consulted a physician. Co-occurrence of migraine increases physician consultations and affects treatment level within the health care system. CAM is also a frequently used treatment option. The chronic headache spectrum seen by GPs and neurologists differs. The high degree of self-management, the high proportion of medication overuse and the frequent use of less well-documented treatment forms is a major concern. Improved management by health professionals, not least GPs, as well as increased use of prophylactic treatment and detoxification of medication overuse is likely to alleviate the burden for those with primary chronic headache. However, those patients who do not seek medical contact also deserve attention.
